# pDNA Impurities in mRNA Vaccines

**DOI:** 10.3390/microorganisms13091975

**Published:** 2025-08-24

**Authors:** Luca Roncati, Nazha Ghaleb, Joya Ghaleb, Karl Kfoury

**Affiliations:** 1 Department of Life Sciences, Health, and Health Care Professions, School of Medicine and Surgery, Link Campus University, 00165 Rome, Italy; 2 Department of Clinical Laboratory Sciences, Faculty of Health Sciences, Balamand University, 00100 Tripoli, Lebanon; 3 Department of Medicine, Faculty of Medicine and Medical Sciences, Balamand University, 00100 Tripoli, Lebanon

Funded during the emergency phase of the severe acute respiratory syndrome coronavirus 2 (SARS-CoV-2) pandemic, messenger RNA (mRNA) vaccines are single-stranded, 5′-capped mRNAs produced using a cell-free in vitro transcription from the corresponding plasmid DNA (pDNA) templates, encoding the viral spike (S) protein of SARS-CoV-2 [1,2]. The expression construct, i.e., the linearized plasmid, is designed to contain regulatory sequences that act as promoter and enhancer regions of the S gene, in order to obtain a significant amount of stable mRNA, which can then be translated into the S protein once inoculated (Figure 1). During the manufacturing process, a nanoscale portion of this pDNA ends up in the vaccine vials in the form of pDNA impurities, which must not exceed a concentration of 10 ηg per dose, as established by the World Health Organization (WHO) [3]. Respecting this limit appears to be of pivotal importance to guarantee high safety standards for human health and thus prevent hypothetical adverse events or reactions.

On 5 August 2025, the U.S. Department of Health and Human Services (HHS) announced the beginning of a coordinated reduction in its mRNA vaccine development activities under the Biomedical Advanced Research and Development Authority (BARDA), including contract termination, de-scoping of mRNA-related work in existing contracts, rejection or cancellation of multiple pre-award solicitations, and restructuring of collaborations [4]. As HHS Secretary Robert F. Kennedy, Jr. stated, this affects 22 projects worth nearly $500 million, while other uses of mRNA technology within the Department have not been impacted [4]. Among the reasons for this announcement is BARDA’s intention to move toward vaccine platforms with stronger safety records, an aspect that has once again brought attention to the potential health risks of mRNA vaccines [5,6,7,8,9].

Although mRNA technology is remarkable and designed to be safe and effective at the same time [10,11,12,13], news of the presence of DNA impurities in mRNA vaccine vials has caused a stir [14]. These impurities, in fact, derive from the pDNA used as a template in the production of the vaccine itself (Figure 1). Some researchers argue that they were at concentrations permitted by the WHO and regulatory authorities (<10 ηg/dose) [15,16], others claim that they were well above the maximum limit even after the purification process [14].

Beyond this controversy [14,15,16,17], questions arise about the impact of these pDNA impurities on health, whether they can trigger innate immunity in genetically predisposed subjects, become incorporated into the DNA of human cells at risk of neoplastic transformation, or evoke an antibody response to some extent while outside the cell. As is known, the human body is able to generate antibodies against double-stranded DNA (dsDNA) or single-stranded DNA (ssDNA), found in numerous rheumatological diseases, particularly systemic lupus erythematosus [18], as well as toxic diffuse goiter and Hashimoto’s thyroiditis [19], diabetes mellitus [20], and autoimmune hepatitis [21]. Since all these diseases have also been reported after the administration of mRNA vaccines against coronavirus disease 2019 (COVID-19) [22,23,24,25,26,27,28,29], further research on this topic is warranted. Just this year, a retrospective study on 98 patients has shown that combining a COVID-19 mRNA vaccine with a COVID-19 vaccine made from inactivated whole viral particles significantly stimulates the production of anti-dsDNA [30], a finding which deserves further investigation on a larger scale.

## Figures and Tables

**Figure 1:**
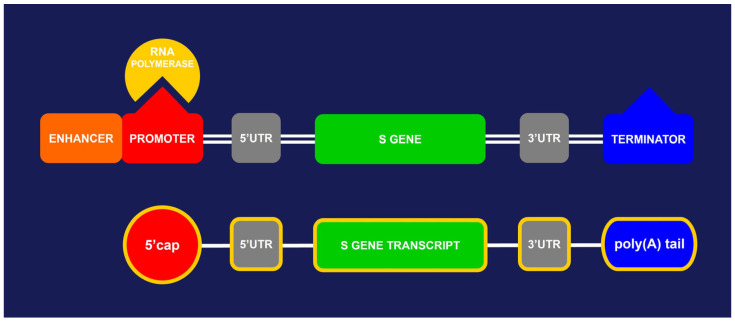
The linearized ds-pDNA used for the synthesis of COVID-19 mRNA vaccines (above) consists of a promoter, to which RNA polymerase binds, an enhancer, two untranslated regions (UTRs) at the 5′ and 3′ ends of the S gene, a transcription terminator, and the gene encoding the S protein of SARS-CoV-2; the corresponding ss-mRNA (below) contains the S gene transcript to be translated into the S protein, the two UTRs, and a 5′ cap plus a poly(A) tail, both introduced by RNA polymerase to protect the transcript from degradation.

## Data Availability

No new data were created or analyzed in this study. Data sharing is not applicable to this article.

## Abbreviations

The following abbreviations are used in this manuscript:

SARS-CoV-2	Severe acute respiratory syndrome coronavirus 2
mRNA	Messenger RNA
pDNA	Plasmid DNA
S	Spike
WHO	World Health Organization
HHS	Health and Human Services
BARDA	Biomedical Advanced Research and Development Authority
dsDNA	Double-stranded DNA
ssDNA	Single-stranded DNA
COVID-19	Coronavirus disease 2019
UTRs	Untranslated regions
